# Biochemical Insights into the Effects of a Small Molecule Drug Candidate on Imatinib-Induced Cardiac Inflammation

**DOI:** 10.3390/ijms26146661

**Published:** 2025-07-11

**Authors:** Renáta Szabó, Denise Börzsei, András Nagy, Viktória Kiss, Zoltán Virág, Gyöngyi Kis, Nikoletta Almási, Szilvia Török, Médea Veszelka, Mária Bagyánszki, Nikolett Bódi, Bence Pál Barta, Patrícia Neuperger, Gabor J. Szebeni, Csaba Varga

**Affiliations:** 1 Department of Physiology, Anatomy, and Neuroscience, Faculty of Science and Informatics, University of Szeged, H-6726 Szeged, Hungary; borzseidenise@gmail.com (D.B.); nagy.andras.levente99@gmail.com (A.N.); k.viktoria007@gmail.com (V.K.); zolee0920@gmail.com (Z.V.); karcsune.kis.gyongyi@szte.hu (G.K.); almasi@expbio.bio.u-szeged.hu (N.A.); tszilvia@bio.u-szeged.hu (S.T.); veszmed@bio.u-szeged.hu (M.V.); bmarcsi@bio.u-szeged.hu (M.B.); bodiniki85@gmail.com (N.B.); bbencep96@gmail.com (B.P.B.); vacs@bio.u-szeged.hu (C.V.); 2 Creative Laboratory Ltd., H-6726 Szeged, Hungary; 3 Laboratory of Functional Genomics, Core Facility, HUN-REN Biological Research Centre, H-6726 Szeged, Hungaryszebeni.gabor@brc.hu (G.J.S.)

**Keywords:** imatinib, inflammation, BGP-15, PARP-1

## Abstract

BGP-15, a poly(ADP-ribose) polymerase-1 (PARP-1) inhibitor exerts cardioprotective effects; however, the underlying mechanisms remain unclear. Therefore, our study aimed to investigate the effects of BGP-15 on the imatinib (Imtb)-induced cardiac inflammation at the biochemical level. Male rats were divided to control, Imtb-treated (60 mg/kg/day for 14 days), and Imtb + BGP-15-treated animals. In this group Imtb was co-administered with BGP-15 at the dose of 10 mg/kg/day. At the end of the experiment, nuclear factor-kappa B/p65 (NF-κB/p65), nuclear transcription factor erythroid-2 related factor (Nrf2), heme oxygenase-1 (HO-1), high mobility group box 1 (HMGB1), and myeloperoxidase (MPO) were measured by Western blot. Chemokine and interleukins (ILs) were determined by Legendplex. Additionally, cardiac specific changes were visualized by immunohistochemistry. We demonstrated that Imtb increased NF-κB/p65, IL-6, IL-1β, IL-18, MCP-1, HMGB1, as well as the expression and activity of MPO. Conversely, the expressions of antioxidant Nrf2 and HO-1 were decreased. Administration of BGP-15 effectively mitigated these inflammatory alterations by significantly reducing pro-inflammatory cytokines and MPO activity, while simultaneously restoring and enhancing the levels of Nrf2 and HO-1, thereby promoting antioxidant defenses. The immunohistochemical staining further supported these biochemical changes. Our study provides new and comprehensive biochemical insight for managing Imtb-induced inflammatory responses via BGP-15-induced PARP1 inhibition.

## 1. Introduction

Cancer is among the leading causes of death worldwide. According to the World Health Organization, an estimated 20 million cancer cases were newly diagnosed in 2022 globally [1]. Although advances in early diagnosis, precise staging and therapy have led to a significant increase in the number of cancer survivors, research into the adverse effects of cancer therapies remains a major challenge for healthcare professionals. Among organ toxicities, cardiovascular side effects are one of the greatest concerns that can occur with many anticancer drugs [2,3]. Imatinib (Imtb) is a selective Bcr-Abl tyrosine kinase inhibitor (TKI) widely used in the treatment of chronic myeloid leukemia (CML), gastrointestinal stromal tumors (GIST), and other malignancies [4]. Despite its clinical efficacy, Imtb has been associated with potential cardiac side effects, primarily mediated by mitochondrial dysfunction, oxidative stress, and endoplasmic reticulum stress pathways, which contribute to cardiomyocyte apoptosis, arrhythmias, or heart failure [5,6]. Although tyrosine kinase inhibitor (TKI)-induced cardiac complications have been demonstrated, further studies are needed to elucidate the underlying mechanisms, with particular emphasis on the roles of oxidative stress and inflammation [7,8,9]. In the chain of oxidative stress-induced deleterious cellular mechanisms, poly(ADP-ribose) polymerase-1 (PARP-1) may play a major role. Inflammatory cascades induced by PARP1 activation, including mitogen-activated protein kinase (MAPK) pathways and nuclear factor κB (NF-κB) [10,11] as well as transcription regulators such as high mobility group box 1 (HMGB1), are major disruptors of cardiovascular homeostasis [12]. Through anti-inflammatory and redox balancing properties, nuclear transcription factor erythroid-2 related factor (Nrf2) serves as a key regulator in cardiovascular homeostasis [13]. Thereby, disruption of the intricate interplay between Nrf2 and NF-κB diminishes transcriptional activation of antioxidant genes, making the heart more vulnerable to injuries.

In this light, the regulation of inflammatory processes has paramount importance in reducing cardiovascular pathologies. To achieve these outcomes, inhibitors of PARP-1 have recently gained considerable interest in cardio-oncology. Among many potential PARP inhibitors, BGP-15 was specifically chosen due to its unique pleiotropic pharmacological profile, characterized by cytoprotective, anti-inflammatory, and oxidative stress-reducing effects. Originally identified during research on heat shock proteins, BGP-15 promotes cellular stress resilience by inducing HSP72 and modulating key signaling pathways involved in inflammation and redox balance. Although its primary effect has been demonstrated against insulin resistance, a growing number of studies also report BGP-15-induced cardioprotective effects [9,14,15,16,17].

While previous studies have focused on the effects of BGP-15 on cardiovascular function in various animal strains and disease models, the aim of our current study was to investigate the effects of BGP-15 in a rat model of Imtb-induced cardiac inflammation at the biochemical level. Given the importance of inflammatory processes and oxidative mechanisms in cardiac side effects, we focused on PARP1-mediated inflammatory pathways. Additionally, we also examined several other inflammation-related biomarkers, including pro-inflammatory cytokines, HMGB1, heme oxygenase-1 (HO-1), and the myeloperoxidase (MPO) enzyme derived from neutrophils.

## 2. Results

### 2.1. Heart Assessment at Necropsy

No pathological macroscopic alterations were observed in the hearts of the rats during necropsy.

### 2.2. Effect of Imatinib and BGP-15-Induced Changes on PARP1 Concentration in the Heart

**Figure 1:**
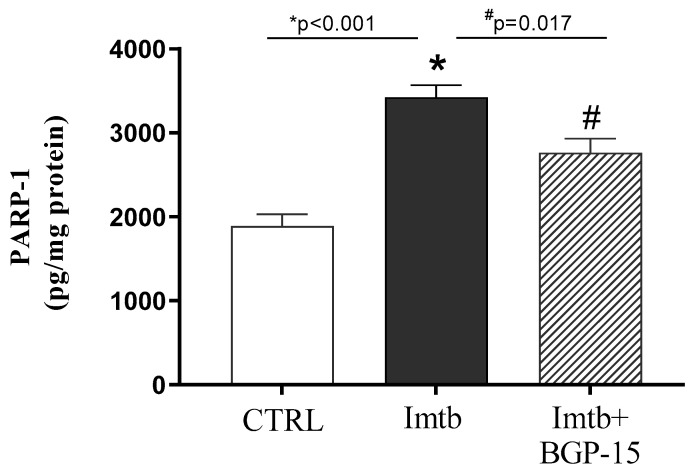
Effect of imatinib and BGP-15-induced changes on the concentration of PARP1 in the heart. Data are represented as the means ± SEMs. n = 7–8. * *p* < 0.05: statistical significance between CTRL and Imtb groups; # *p* < 0.05: statistical significance between Imtb and Imtb + BGP-15 groups. PARP1: Poly(ADP-ribose) polymerase-1, CTRL: control, Imtb: imatinib.

To demonstrate that PARP1 plays a major role in Imtb-mediated mechanisms and is a key target of BGP-15, PARP1 concentrations were determined. Our results clearly show that the chemotherapeutic agent, Imtb, resulted in a 1.75-fold increase in PARP1 concentration compared to the CTRL group; however, BGP-15 treatment effectively diminished the Imtb-induced adverse changes. The data are presented in Figure 1.

### 2.3. Effect of Imatinib and BGP-15-Induced Changes on the Expressions of NF-κB/p65 and Nrf2 in the Heart

**Figure 2:**
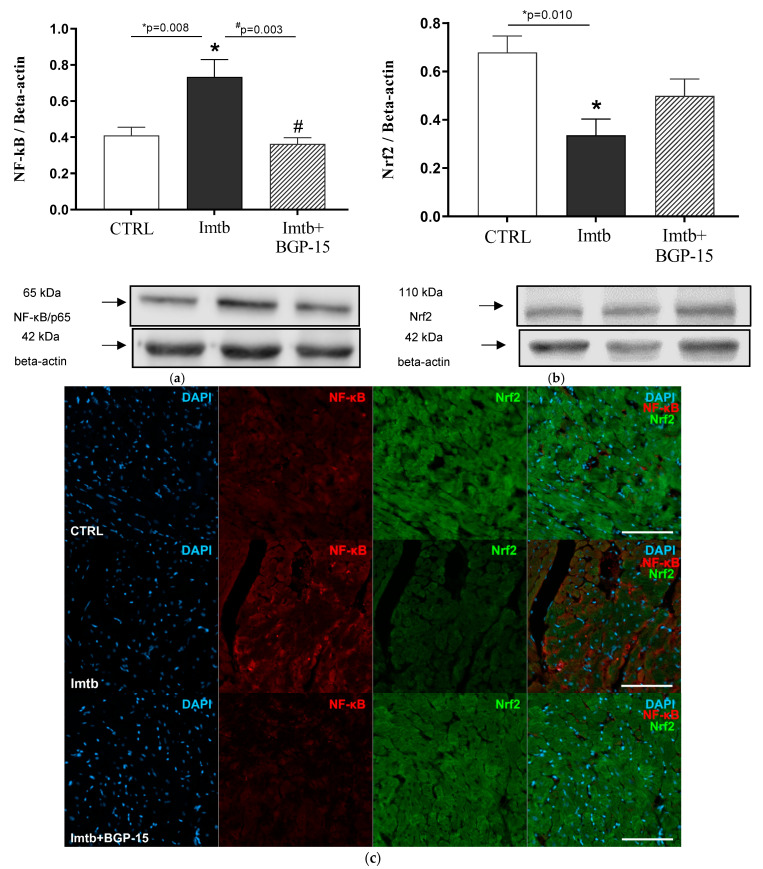
Effect of imatinib and BGP-15-induced changes on the expressions of (**a**): NF-κB/p65 and (**b**): Nrf2 in the heart. (**c**): Representative fluorescent micrographs illustrate NFκB/p65 (red) and Nrf2 (green) staining in rat ventricular myocardium. Nuclei were counterstained with DAPI (blue). Scale bars indicate 100 µm. Data are represented as the means ± SEMs. n = 6–7 for NF-κB/p65 and n = 6–7 for Nrf2. * *p* < 0.05: statistical significance between CTRL and Imtb groups; # *p* < 0.05: statistical significance between Imtb and Imtb + BGP-15 groups. NF-κB/p65: nuclear factor κB/p65 domain, Nrf2: nuclear transcription factor erythroid-2 related factor, CTRL: control, Imtb: imatinib.

To confirm the role of NF-κB/p65 in Imtb- and BGP-15-mediated effects, changes in p65 transactivation domain expression were assessed. As shown in Figure 2a, 2 weeks of Imtb administration significantly increased the expression of NF-κB/p65 in the heart, which was mitigated by BGP-15. BGP-15 treatment reduced NF-κB/p65 expression to the levels seen in the CTRL group. Cross-talk between NF-κB/p65 and Nrf2 is well-established in drug-induced toxicities; thereby, we next determined how Nrf2 expression was altered in Imtb-treated rat hearts. In contrast to NF-κB/p65, Nrf2 expression significantly decreased as a result of Imtb-induced toxicity; however, BGP-15 therapy was able to ameliorate Nrf2 expression. The opposite changes in NF-κB/p65 and Nrf2 levels suggest that an interplay exists between the two factors. The changes in Nrf2 expression are shown in Figure 2b. Immunohistochemistry was performed to visualize the cardiac expression of NF-κB/p65 and Nrf2 in the heart. The dual-labeled fluorescent staining demonstrated immunoreactivity consistent with protein expression levels detected by Western blot analysis for both NF-κB/p65 and Nrf2 in ventricular myocardium in all three experimental groups. Representative images are shown in Figure 2c.

### 2.4. Effect of Imatinib and BGP-15-Induced Changes on HO-1 Expression in the Heart

**Figure 3:**
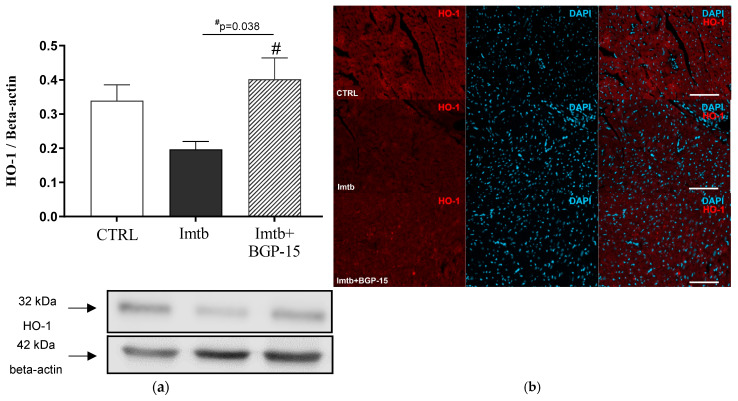
Effect of imatinib and BGP-15-induced changes on the expression of HO-1 in the heart (**a**). (**b**): Representative fluorescent micrographs illustrate HO-1 (red) staining in rat ventricular myocardium. Nuclei were counterstained with DAPI (blue). Scale bars indicate 100 µm. Data are represented as the means ± SEMs. n = 6–7. # *p* < 0.05: statistical significance between Imtb and Imtb + BGP-15 groups. HO-1: heme oxygenase-1, CTRL: control, Imtb: imatinib.

HO-1 is one of the antioxidant genes, which is regulated through the Nrf2 pathway. To determine whether BGP-15-induced amelioration of Nrf2 expression contributes to antioxidant mechanisms, cardiac HO-1 expression was also evaluated. Similar to Nrf2 expression changes, 2 weeks of Imtb treatment decreased the HO-1 expression, whereas BGP-15 caused an enhancement. The data are presented in Figure 3a. In addition, as shown in Figure 3b, the HO-1 immunoreactivity in the rat heart ventricular myocardium reflected the abovementioned changes among the three treatment groups.

### 2.5. Effect of Imatinib and BGP-15-Induced Changes on Inflammatory Cytokines in the Heart

**Figure 4:**
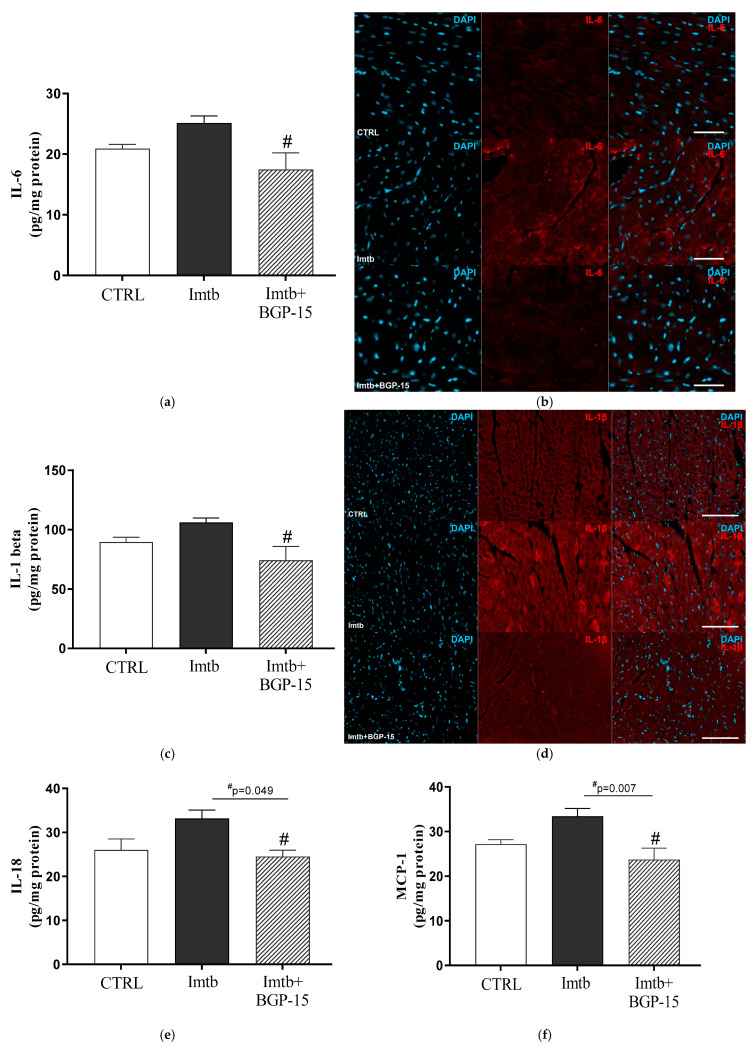
Effect of imatinib and BGP-15-induced changes on the concentrations of pro-inflammatory cytokines in the heart. (**a**): IL-6 concentration, (**b**): representative fluorescent micrographs illustrate IL-6 (red) staining in rat ventricular myocardium. Nuclei were counterstained with DAPI (blue). Scale bars indicate 50 µm. (**c**): IL-1β concentration, (**d**): representative fluorescent micrographs illustrate IL-1β (red) staining in rat ventricular myocardium. Nuclei were counterstained with DAPI (blue). Scale bars indicate 100 µm. (**e**): IL-18 concentration, (**f**): MCP-1 concentration. Data are represented as the means ± SEMs. n = 5–6. # *p* < 0.05: statistical significance between Imtb and Imtb + BGP-15 groups. IL-6: interleukin-6, IL-1β: interleukin-1beta, IL-18: interleukin-18, MCP-1: monocyte chemoattractant protein-1, CTRL: control, Imtb: imatinib.

At the end of the experimental period, concentrations of key pro-inflammatory cytokines, including IL-6, IL-1β, IL-18, and MCP-1 were measured. Figure 4a–f demonstrate that Imtb increased the cardiac concentrations of these cytokines; however, a significant improvement was observed following the BGP-15 treatment. Four weeks of BGP-15 administration resulted in a significant decrease in the concentration of each cytokine compared to the Imtb-treated group. The immunohistochemical analysis carried out on rat ventricular myocardium with IL-6 and IL-1β staining revealed similar pattern of changes between the groups (Figure 4b,d).

### 2.6. Effect of Imatinib and BGP-15-Induced Changes on HMGB1 Expression in the Heart

**Figure 5:**
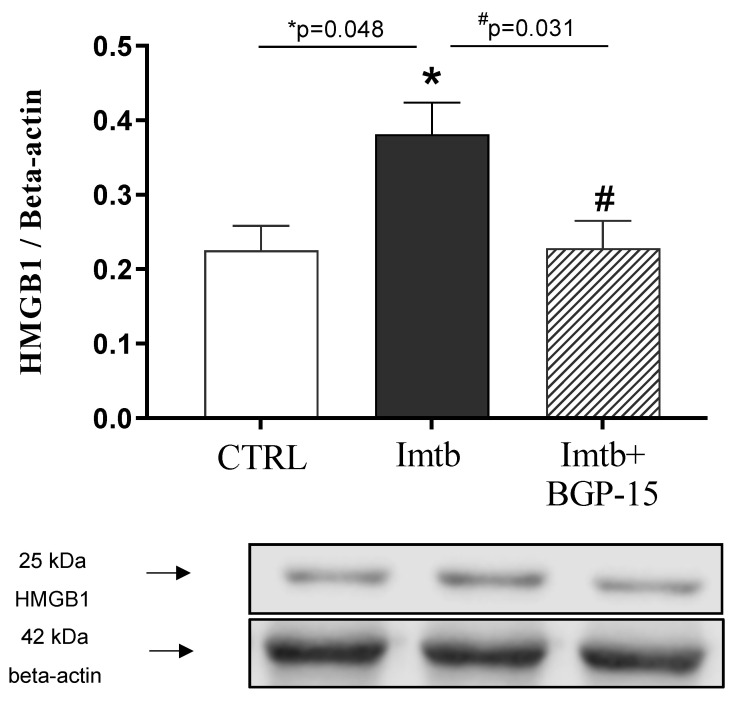
Effect of imatinib and BGP-15-induced changes on the expression of HMGB1 in the heart. Data are represented as the means ± SEMs. n = 5–8. * *p* < 0.05: statistical significance between CTRL and Imtb groups; * *p* < 0.05: statistical significance between CTRL and Imtb groups; # *p* < 0.05: statistical significance between Imtb and Imtb + BGP-15 groups. HMGB1: high mobility group box 1, CTRL: control, Imtb: imatinib.

To determine whether HMGB1 was related to Imtb-induced inflammatory state, cardiac HMGB1 expression was evaluated. As shown in Figure 5, Western blot analysis revealed that Imtb administration led to a significant increase in HMGB1 level. However, the BGP-15 treatment proved to be effective as BGP-15 halved the values increased by Imtb.

### 2.7. Effect of Imatinib and BGP-15-Induced Changes on the Expression and Activity of MPO in the Heart

**Figure 6:**
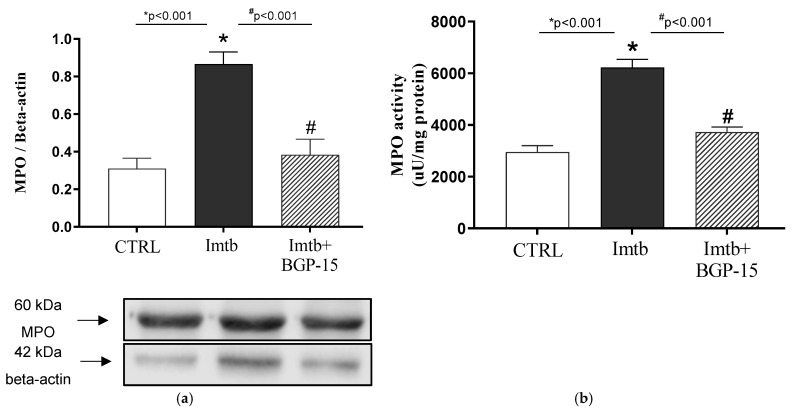
Effect of imatinib and BGP-15-induced changes on (**a**): the expression and (**b**): activity of MPO enzyme in the heart. Data are represented as the means ± SEMs. n = 5–8 for MPO expression and n = 6–8 for MPO activity. * *p* < 0.05: statistical significance between CTRL and Imtb groups; * *p* < 0.05: statistical significance between CTRL and Imtb groups; # *p* < 0.05: statistical significance between Imtb and Imtb + BGP-15 groups. MPO: myeloperoxidase, CTRL: control, Imtb: imatinib.

It has been demonstrated that Imtb induced inflammatory mechanisms via NF-κB-mediated pro-inflammatory cytokine release, whereas BGP-15 has anti-inflammatory properties. To assess the role of inflammatory mediators in neutrophil recruitment and thus in the potential inflammatory state, the expression and activity of MPO enzyme were determined. As expected, Imtb resulted in a significant increase in MPO expression; however, this pathological level was ameliorated by BGP-15 (Figure 6a). Our results show that changes in MPO activity are consistent with changes in MPO expression. Figure 6b demonstrates that MPO activity is more than two-folds higher in Imtb-treated animals compared to the CTRL group. BGP-15 has effectively reduced this unfavorable, high activity value.

## 3. Discussion

Over the past few decades, the development of new cancer therapies has dramatically changed the landscape of treatment approaches for several malignancies; however, the emergence of adverse side effects has also come to the fore. Cardiovascular side effects are the epitome of such concerns, which acutely or chronically affects the prognosis and life quality of cancer patients and survivors. Although TKI-induced cardiac damage to myocardial structure and function has been documented [18], the molecular mechanisms behind these events are not fully elucidated. Therefore, understanding the underlying mechanisms and identifying effective treatments are major health challenges. In our current work, the effects of BGP-15, a small molecule PARP inhibitor, were examined in a rat model of Imtb-induced inflammation.

In this model, rats were subjected to short-term, low-dose administration of imatinib (60 mg/kg/day for 14 consecutive days), which was chosen based on the prescribing information for Gleevec [19] and previous studies [20,21,22]. Kerkelä et al. found that a low dose of imatinib (50 mg/kg for 3 weeks) has been associated with ultrastructure changes, mitochondrial abnormalities, and recruitment of ER stress response [22]. Similarly, Herman et al. proved that low-dose imatinib treatment for 14 days resulted in cardiotoxic manifestation [21]. Detailed examination of the multiple mechanisms that potentially contribute to imatinib-induced cardiotoxicity is necessary for therapeutic interventions. Although the correlation between inflammation and cancer, best described as perpetual induction, is well established, a growing body of evidence suggests that oxidative stress and inflammation are closely associated with drug-induced cardiac side effects. In case of the relationship between cancer and inflammation, inflammatory processes create a microenvironment in which DNA mutation rates, proliferation, and cell death are less regulated. In turn, cancer cells promote inflammatory processes by directly recruiting neutrophils and macrophages or indirectly, by damaging the surrounding tissue which results in inflammatory responses [23]. Regarding chemotherapeutic agents, inflammation often appears as a major side effect during treatment [24]. Chemotherapy-induced inflammation might affect multiple organs and organ systems, including kidneys, the gut, the liver, the central nervous system [25], and most notably the heart, where inflammation represents a central cause for drug-induced cardiotoxicity. The underlying mechanisms of chemotherapy-associated inflammation are complex and multifactorial. In this complex regulatory network, mitochondrial dysfunction and the related oxidative stress may serve as a major trigger for PARP activation [26]. PARPs play an important role in oxidative stress and inflammation-induced mechanisms at three levels: chromatin remodeling regulation, transcriptional activity regulation, and mRNA posttranscriptional stability regulation [27]. PARP1-related functions are mediated through Poly ADP-Ribosylation (PARylation), which is induced by cellular stress or DNA damage and affects inflammatory processes. PARylation can modulate the functions of target proteins by modifying their binding affinity to their DNA/RNA partners [28]. Previous studies proved that NF-κB is among the acceptor proteins of PARylation; thus, PARP1 serves a co-activator of NF-κB [29,30]. The classical NF-κB heterodimer consists of a p65 transactivation domain and a p50 subunit, which serves as a helper in NF-κB-DNA binding [31]. As a result of PARylation, a transactivation-competent complex is assembled, followed by nuclear retention of NF-κB/p65 and results in up-regulation of various pro-inflammatory cytokines [29,32]. Supporting this molecular background, our results show that imatinib treatment significantly increased cardiac PARP1 concentration and NF-κB/p65 expression, accompanied by elevated pro-inflammatory cytokine levels, including IL-6, IL-1β, IL-18, and MCP-1. In agreement with our findings, Zerfaoui et al. also demonstrated that PARP1 expression is associated with dysregulated NF-κB activity, which can contribute to cellular dysfunction and increased inflammatory responses [33]. In light of this direct relationship, the inhibition of PARP1 and the consequent blockade of NF-κB-related pathways are key targets for reducing inflammatory conditions. Many pharmacological inhibitors of PARP have been developed over the past decades. They play a major role not only in cancer therapies, but also in various cardiovascular disorders associated with acute or chronic inflammation [34,35]. Luo et al. demonstrated that the suppression of PARP1 inhibited the NF-κB pathway, decreased the production of inflammatory cytokines and thus improved cardiac function [30]. In another model, Eid et al. also found that the inhibition of the PARP1/ NF-κB axis ameliorated cardiovascular pathologies [36]. Considering all these findings, PARP1 inhibition represents a promising approach to reduce chemotherapy-induced pathologies.

In this present study, a small molecule BGP-15 (C_14_H_22_N_4_O_2_·2HCl) was selected to examine its potential anti-inflammatory role. BGP-15 was originally developed by N-Gene Research Laboratories Inc. and exhibits pleiotropic pharmacodynamics effects, including cytoprotection, metabolic regulation, and oxidative stress-reducing action. It has been shown that BGP-15 induces heat shock proteins (HSPs), particularly HSP72, which promotes cellular stress resilience [16]. Additionally, BGP-15 suppresses the c-Jun N-terminal kinase, which is implicated in insulin resistance as well as metabolic disorders [37,38], and inhibits PARP1enzyme, thereby reducing oxidative stress-induced cell damage. The effects of BGP-15 have been well-documented in various animal models and experimental conditions, which provide important context for our findings. For instance, the anti-inflammatory effects of BGP-15 have been demonstrated in aging ZDF rats [39] and spontaneously hypertensive rats (SHR) with a specific focus on mitochondrial function [15]. BGP-15 has been shown to exert indirect PARP inhibition through its stabilizing effects on mitochondrial function. As demonstrated by Sumegi et al. [40], BGP-15 accumulates in mitochondria where it prevents membrane depolarization and preserves the normal activity of complex I and complex III. This leads to a reduction in mitochondrial reactive oxygen species (ROS) production, thereby decreasing oxidative damage and subsequent PARP activation. Additionally, BGP-15 indirectly contributes to mitochondrial stability by modulating pro- and anti-apoptotic signaling pathways, further supporting its protective role. Additionally, in Langendorff ex vivo heart perfusion system, Szabados et al. found that BGP-15 decreased oxidative stress and confirmed that this effect was mediated by direct inhibition of PARP. This result was tested on isolated enzyme and the kinetic analysis demonstrated a mixed-type (noncompetitive) inhibition with K(i) = 57 ± 6 µM [41]. Moreover, BGP-15 has also been shown to participate in indirect PARP inhibition due to its stabilizing effect on mitochondrial function. In further experiments, Sarszegi et al. investigated the effects of BGP-15 on imatinib-induced cardiotoxicity, focusing on MAP kinases and the phosphorylation of Akt and GSK-3beta. They found that imatinib significantly increased PARP expression; however, BGP-15 effectively suppressed adverse changes in isolated perfused rat heart [9]. These previous studies provide a broader perspective on the cardiovascular and cellular effects of BGP-15. However, most of them did not explore in detail the biochemical and molecular pathways specifically linked to inflammation. Our work aims to contribute to this gap by addressing these mechanisms at the biochemical level. Our results are consistent with findings from previous models, providing further evidence that BGP-15 contributes to the attenuation of oxidative stress and inflammation, offering new insights into its role in cardio-oncology. We found that BGP-15 treatment resulted in a beneficial effect on the PARP1/ NF-κB axis that was verified by the reduction in pro-inflammatory cytokine and chemokine levels.

In addition to NF-κB, Nrf2 is a crucial target that modulates cellular redox status and inflammatory responses. A broad range of studies has confirmed the functional interplay between NF-κB and Nrf2 as they can regulate their expression in a complex regulatory loop [13,42,43]. In a recent work, Casper provided insight into the cross-talk between NF-κB and Nrf2 and showed that NF-κB transcription inhibits Nrf2 activation by reducing ARE gene transcription, while the Nrf2 pathway can decrease the level of active NF-κB by enhancing antioxidant defense mechanisms to scavenge reactive oxygen species [44]. As a result of Nrf2 activation, a conformational change triggers Nrf2 translocation into the nucleus, where it heterodimerizes with Maf proteins and binds to the ARE target sequence in DNA [45]. In addition to the highly connected interplay between NF-κB and Nrf2, the role of PARP1 further increases the complexity of the interactions. PARP1 both serves as trigger for the translocation of NF-κB to the nucleus and a direct transactivator role in NF-κB-mediated transcription; therefore, PARP-1 mediated NF-κB has an impact on Nrf2. Consequently, PARP-1 may have an indirect effect on Nrf2 via PARP1-mediated NF-κB activation. Our experiment provides an insight into a sub-process of this complex regulatory network. As a result of imatinib administration, Nrf2 expression is significantly decreased, which was ameliorated by BGP-15 treatment. These changes are consistent with those observed for PARP1 concentration and NF-κB/p65 expression. Similar to changes in Nrf2 expression, two weeks of imatinib administration reduced cardiac HO-1 expression, which was enhanced by BGP-15 administration. Among antioxidant genes, HO-1 is enhanced by Nrf2 and serves as a major regulator in the maintenance of redox balance [46]. Induction of HO-1 can confer protection against inflammatory conditions through the removal of pro-oxidant heme, whereas heme-derived reaction products, including biliverdin, bilirubin, and carbon monoxide, contribute HO-dependent antioxidant, anti-inflammatory, anti-apoptotic, and anti-proliferative effects in the cardiovascular system [47,48].

Regarding the complex inflammatory pathways, PARP1-mediated PARylation may also affect the release of HMGB1 into the cytoplasm. HMGB1 acts as a DNA-binding protein and can also be passively translocated into the cytoplasm as a result of NETosis, pyroptosis, ferroptosis, etc. In the cytoplasm, HMGB1 serves as a damage-associated molecular pattern (DAMP) molecule, primarily inducing inflammatory responses, including NF-κB activation [49]. HMGB1 is a crucial biomarker in inflammation-related diseases, particularly in cardiovascular pathologies. Previously, Yuan et al. summarized that HMGB1 release can be associated with chemotherapy- and drug-induced cardiac damage [50]. It has been shown that HMGB1 is a major contributor to the second-generation TKI dasatinib-induced cardiotoxicity [51]. Tang et al. examined the role of imatinib-induced cardiac injury in a rat cardiomyocyte cell line and found that HMGB1 is a major target in cardiac damage [52].

**Figure 7:**
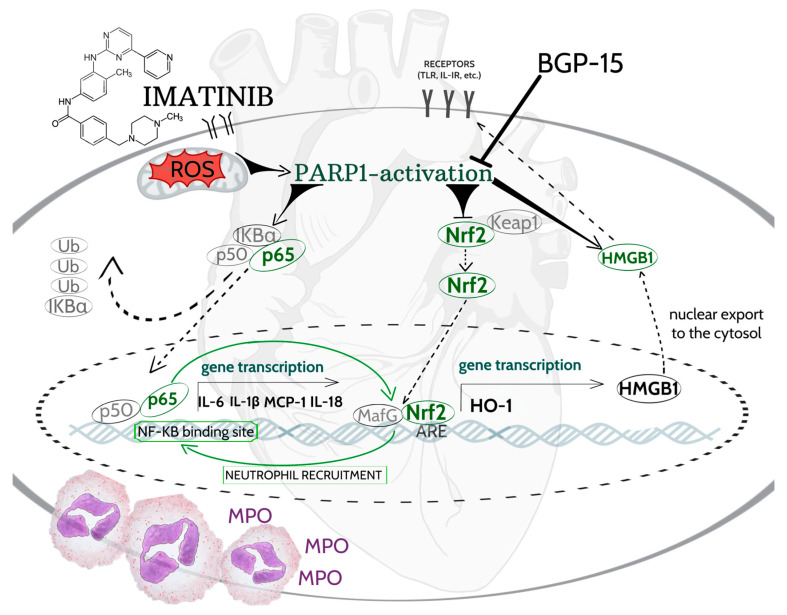
PARP1 plays a major role in imatinib-mediated inflammatory processes and also serves as a key target of BGP-15. Imatinib-induced mitochondrial damage results in ROS accumulation, which induces PARP1 activation. PARP1 augments NF-κB-mediated gene expression by its nuclear coactivator function; thus, pro-inflammatory genes, such as IL-6, IL-18, MCP-1, and IL-1β are upregulated. IL-1β and IL-18 and play a role in immune response by recruiting and activating neutrophils. Thus, neutrophil infiltration and inflammation can be associated with increased MPO level. There is a strongly connected interplay between NF-κB and Nrf2. As a consequence of NF-κB activation, Nrf2-dependent HO-1 gene transcription is down-regulated, which decreases the antioxidant defense mechanisms. Furthermore, PARP1 catalyzes PARylation of HMGB1, which facilitates its dissociation from chromatin and translocation to cytosol. Cytoplasmic HMGB1 may bind to receptors, such as TLR, and further augment the downstream NF-κB signaling. In contrast to the imatinib-induced effects, BGP-15, a small molecule drug candidate, is able to reduce inflammatory responses through the inhibition of PARP1. NF-κB: nuclear factor-κappa B, Nrf2: nuclear transcription factor erythroid-2 related factor, HMGB1: high mobility group box 1, IL-6: interleukin-6, IL-1β: interleukin-1beta, IL-18: interleukin-18, MCP-1: monocyte chemoattractant protein-1, HO: heme oxygenase, MPO: myeloperoxidase, ARE: antioxidant response element.

Previous studies have shown that oxidative stress-related pathological conditions increase neutrophil infiltration, which is also observed in cardiovascular injuries. The underlying mechanisms can be associated with the interaction of DAMP and toll-like receptors (TLRs), which are key mediators of the innate immune system’s inflammatory response. Consequently, the activation of TLRs leads to nuclear translocation of NF-κB and downstream transcription of inflammatory cytokines and chemokine, including IL-1β, IL-18, and MCP-1, which help recruit neutrophils. It also exacerbates inflammation and contributes to cardiac damage [53]. Neutrophil-driven inflammation can be confirmed by the detection of biomarkers, which include MPO. The activation of MPO generates hypchlorous acid (HOCl) production via catalyzing the reaction of chloride and H_2_O_2_, which further exacerbate inflammation and impair redox homeostasis [54]. Furthermore, new research findings highlight that MPO can act as a bridge linking oxidative stress and inflammation to cardiac pathologies [55,56,57]. Our current findings show that administration of 2 weeks of imatinib resulted in an increased MPO activity and expression associated with NF-κB-mediated inflammatory responses. However, PARP inhibition by BGP-15 effectively attenuated the elevated MPO levels. Figure 7 summarizes the BGP-15-mediated target pathways in imatinib-induced cardiac inflammation.

### Limitations

In the present study, we focused exclusively on biochemical measurements to investigate the effects of BGP-15 on Imtb-induced cardiac inflammation. While these data provide valuable mechanistic insights, it is important to acknowledge certain limitations. Our experimental design did not include echocardiographic or histological analyses, which would have allowed functional and structural assessment of cardiac changes. Future studies incorporating these methods could provide a more comprehensive understanding of the cardioprotective effects of BGP-15 and correlate biochemical findings with histological and functional outcomes.

## 4. Materials and Methods

### 4.1. Animals and Experimental Protocol

In all procedures the EU directive 2010/63/EU for the protection of animals used for scientific purposes were strictly followed, and all the experiments were approved by Animal Welfare Committee University of Szeged with the license number XX./3205/2022. Twelve-week-old male Wistar rats were purchased from Animalab Ltd., Vác, Hungary. According to the Directive 2010/63/EU, the rats were housed at a temperature of 20–23 °C with a daily 12 h light/dark cycle and were given a standard diet and tap water ad libitum. Thirty-three rats were randomly divided into three groups. The first group (total n = 11/group) was designed as a control (CTRL) group. To investigate the underlying mechanisms of cardiac inflammation, animals in the second group (total n = 12/group) received a low dose of imatinib (Imtb, Sandoz) at a daily dose of 60 mg/kg per os for 2 weeks. The dose of Imtb was selected to align with the clinically established dosing range of 400–800 mg/day for a 70 kg adult, as applied in therapeutic protocols [58,59]. In the third group (total n = 9/group), imatinib (60 mg/kg daily once per os for 14 consecutive days) was co-administered with BGP-15 (HY-100828, MedChemExpress, Monmouth Junction, NJ, USA) at the dose of 10 mg/kg once daily per os. For this group, imatinib and BGP-15 treatments were started at the same time; however, BGP-15 administration continued for an additional 2 weeks. Preclinical data suggest that administration of BGP-15 may support translational value of inflammation-related cardiac pathologies [15,16]. On the day following the last dose of BGP-15, rats from all three groups were sacrificed using intraperitoneal injection of thiopental (100 mg/kg, B. Braun Medical SA, Barcelona, Spain) and the hearts were excised. One subset of the excised hearts was placed at −80 °C for further biochemical measurements. To support our biochemical results, the other subset of the hearts was fixed in paraformaldehyde (4% in phosphate-buffered saline) and embedded in paraffin for representative immunofluorescence-staining.

### 4.2. Measurement of Cardiac PARP1 Concentration

At the end of the experimental period, PARP1 concentration was measured from cardiac tissue. To determine PARP1 concentration, tissue samples were homogenized (Benchmark D1000 Homogenizer, Sayreville, NJ, USA, 2× *g* 10 s) in ice-cold phosphate buffer (pH 7.4) and then centrifuged at 5000× *g* for 5 min at 4 °C. After centrifugation, sample supernatants were assayed with enzyme-linked immunosorbent assay (ELISA) kit purchased from FineTest (Wuhan, China). Briefly, 100 µL standard or sample were added to each well of the microplate, incubated for 90 min at 37 °C, and then washed twice. This was followed by the addition of 100 µL biotin-labeled antibody working solution and the microplate was incubated for 30 min at 37 °C. For color development, 90 µL 3,3′,5,5′-Tetramethylbenzidine (TMB) substrate was added to each well, followed by a 15 min incubation at 37 °C. Finally, the reaction was stopped with 50 µL of the stop solution, and absorbance was measured immediately at 450 nm (Benchmark Microplate reader, Bio-Rad, Hercules, CA, USA). Cardiac PARP1 concentration was expressed as pg/mg protein.

### 4.3. Western Blotting of NF-κB/p65, Nrf2, HO-1, HMGB1, and MPO

Cardiac samples were homogenized in RIPA buffer (Merck Millipore, Burlington, MA, USA) three 10 s intervals using Ultrasonic Homogenizer UP-100H (Hielscher Ultrasonics, Teltow, Germany). The samples were then centrifuged at 15,000× *g* for 10 min at 4 °C, and the supernatants were collected. Protein concentration was determined using a Pierce Bicinchoninic Acid (BCA) protein assay kit (Pierce, Thermo Fisher Scientific, Waltham, MA, USA). Based on the protein concentration, equal amounts (50 µg) from each sample were calculated and loaded onto 10% sodium dodecyl sulfate (SDS)-polyacrylamide gels (90 V, 2 h). Proteins were transferred onto nitrocellulose membranes (35 V, 2.5 h), dyed with Ponceau solution and washed in TBS-Tween (TBS-T, pH 7.4). Subsequently, nonspecific binding was blocked by overnight incubation with 5% nonfat dry milk powder or 5% bovine serum albumin (BSA) dissolved in TBS-T. Afterwards, blots were washed three times for 10 min in TBS-T and incubated with the primary antibodies for 2 h at room temperature: anti- NF-κB/p65 (1:500, ab16502, Abcam, Cambridge, UK), anti-Nrf2 (1:500, 16396-1-AP, Proteintech, Manchester, UK), anti-HMGB1 (1:1000, ab79823, Abcam, Cambridge, UK), anti-HO-1 (1:250, ADI-OSA-111, Enzo Life Sciences, Farmingdale, NY, USA) as well as anti-MPO (1:1000, ab208670, Abcam, Cambridge, UK). Incubation with either polyclonal goat anti-rabbit (1:5000, Dako Agilent, Santa Clara, CA, USA) for NF-κB/p65, Nrf2, HMGB1, and MPO, or polyclonal rabbit anti-mouse antibodies conjugated with horseradish peroxidase (1:5000, Dako Agilent, Santa Clara, CA, USA) for HO-1 was carried out for 1 h at room temperature. For immunoblots, *β*-actin served as the loading control to show that similar amounts of protein were loaded in each lane. The membranes were incubated with anti-*β*-actin primary antibody (1:10,000, ab20272, Abcam, Cambridge, UK) for 2 h at room temperature and polyclonal rabbit anti-mouse secondary antibodies conjugated with horseradish peroxidase (1:5000, Dako Agilent, Santa Clara, CA, USA).

MagicMark XP Western Protein Standard (Invitrogen, Thermo Fisher Scientific, Waltham, MA, USA) was used for convenient protein determination. The standard consists of 9 recombinant proteins ranging in molecular weight from 20 to 220 kDa allowing the accurate identification of the proteins [60]. The bands were visualized by Li-Cor Odyssey XF imaging system (LiCorbio, Lincoln, NE, USA), developed using an enhanced chemiluminescence system (ECL Plus, Amersham Pharmacia Biotech., Buckinghamshire, UK), and analyzed using Quantity One Software version 4.5 (Bio-Rad Laboratories, Hercules, CA, USA). Results were normalized to β-actin and presented as relative expressions.

### 4.4. Determination of Cardiac Inflammatory Cytokines

Cardiac samples were homogenized in RIPA buffer (Merck Millipore, Burlington, MA, USA) three times on ice for 10 sec using Ultrasonic Homogenizer UP-100H (Hielscher Ultrasonics, Teltow, Germany). Following centrifugation at 15,000× *g* for 10 min at 4 °C, the supernatant fraction was collected. Legendplex technology, a multiplex bead-based immunoassay (Rat Inflammation Panel, cat n.: 740401, Biolegend, San Diego, CA, USA), was used to determine the concentrations of IL-1β, IL-6, IL-18, and MCP-1 following the instructions of the manufacturer. Briefly, tissue homogenates were incubated with premixed capture beads, followed by washing, and the addition of detection antibodies. After washing, streptavidin-PE was finally added. A standard curve was generated by the application of the commercial cytokine standards of the kit. Detection range was from 20 ng/mL to 4.8 pg/mL for IL-6; from 50ng/mL to 12.2 pg/mL for MCP-1, IL-18, and IL-1β. Samples were acquired on a Cytoflex S FACS (Beckman Coulter, Budapest, Hungary) using the APC and PE channels. Evaluation was performed in CytExpert 4.0 (Beckman Coultrer) and Microsoft Excel fitting the median values to the curve of the standards [61]. The whole protein concentration was measured using a BCA protein assay kit. The level of the cytokine/chemokine values were calculated in relation to the whole protein concentration and expressed as pg/mg protein.

### 4.5. Measurement of Cardiac MPO Activity

Cardiac tissue samples were homogenized (Benchmark D1000 Homogenizer) for 2× 10 s in a PBS and 0.5% hexadecyltrimethylammonium bromide (HETAB) solution. The samples were frozen and thawed four times, followed by 15 min of centrifugation at 10,000× *g* and 4 °C. An amount of 12 µL of standard or sample supernatants were added to 280 µL *o*-dianisidine dihydrochloride, using a 96-well plate. Finally, the reaction was initiated by adding 20 µL hydrogen peroxide to each well. The mixture was shaken for 30 s, and MPO activity was measured spectrophotometrically at 490 nm (Benchmark Microplate reader, Bio-Rad, Hercules, CA, USA) [60]. The obtained values were expressed as µU/mg protein.

### 4.6. Fluorescent Immunohistochemistry

For immunofluorescence studies, IL-1β, IL-6, or HO-1 single-labeling and NF-κB/p65-Nrf2 double labeling immunohistochemistry were performed on heart paraffin sections (5 µm) from each experimental group. After blocking in tris(hydroxymethyl)aminomethane-buffered saline (TBS) containing 1% bovine serum albumin and 10% normal goat serum, the sections were incubated overnight at 4 °C with primary antibodies (NF-κB/p65: 1:50, sc-8008, Santa Cruz Biotechnology, Dallas, TX, USA; Nrf2: 1:100, ab31163, Abcam, Cambridge, UK; IL-1β: 1:50, sc52012, Santa Cruz Biotechnology, USA; IL-6: 1:50, ab9324, Abcam, Cambridge, UK; HO-1: 1:200, NBP1-97507, Novus Biologicals, Centennial, CO, USA). After washing in TBS with 0.025% Triton X-100, sections were incubated with anti-mouse Cy^TM^3 (1:200; Jackson ImmunoResearch Laboratories, Pennsylvania, PA, USA; Cat. No. 115-165-003) and anti-rabbit Alexa Fluor 488 (1:200; Invitrogen, Thermo Fisher Scientific, Waltham, MA, USA; Cat. No. A11008; in the case of double-labeling) secondary antibodies for 1 h at room temperature. All immunostained sections were counterstained with DAPI and mounted with Fluoromount^TM^ Aqueous Mounting Medium (Sigma–Aldrich, Budapest, Hungary). Representative photographs were visualized using Zeiss Imager Z.2 fluorescent microscope equipped with an Axiocam 506 mono camera (Zeiss, Oberkochen, Germany) [62].

### 4.7. Statistical Analysis

All data are presented as mean ± SEM. Statistical analysis was carried out with SigmaPlot 12 software, (Systat Software Inc., San Jose, CA, USA). Normality was checked in all datasets with the Shapiro–Wilk test. For parametric distribution, one-way ANOVA followed by Tukey post hoc test was used, and the Kruskal–Wallis test followed by Dunn’s method was chosen in the case of nonparametric data. Differences were considered significant in all measurements when the *p* values were less than 0.05.

## 5. Conclusions

Our results show that PARP activation plays a major role in the inflammatory processes, whereas BGP-15, a small molecule PARP inhibitor, mitigated imatinib-related adverse changes. Examination of the underlying mechanisms confirmed that PARP1-mediated inflammation is driven by the modulation of NF-κB/Nrf2-related mechanisms and by influencing HMGB1 expression. Targeting these mechanisms, BGP-15 is an effective therapeutic molecule to counteract imatinib-induced pathological changes.

Our results provide a comprehensive insight into PARP1-mediated inflammatory mechanisms at the biochemical level. For a better understanding of BGP-15-induced PARP1 inhibition in imatinib-induced cardiac pathologies, more detailed molecular analysis is a major priority for future objectives.

## Data Availability

Data will be made available on request.

## Abbreviations

The following abbreviations are used in this manuscript:

PARP-1	poly(ADP-ribose) polymerase-1
Imtb	imatinib
NF-κB/p65	nuclear factor-kappa B/p65
Nrf2	nuclear transcription factor erythroid-2 related factor
HO-1	heme oxygenase-1
HMGB 1	high mobility group box 1
MPO	myeloperoxidase
IL	interleukin
